# Tacrolimus-induced reversible posterior leukoencephalopathy syndrome in a child with nephrotic syndrome: a case report

**DOI:** 10.3389/fped.2026.1690215

**Published:** 2026-01-29

**Authors:** Dengyan Wu, Lina Ma, Huimin Wu, Jie Li, Jing Yang, Li Huang

**Affiliations:** 1 Department of Pediatric Nephrology, Lanzhou University Second Hospital, Lanzhou, China; 2 Department of Nephrology, Gansu Province Children’s Hospital, Lanzhou, China; 3 Department of Nuclear Magnetic Resonance, Lanzhou University Second Hospital, Lanzhou, China

**Keywords:** adverse effects, children, nephrotic syndrome, reversible posterior leukoencephalopathy syndrome, tacrolimus

## Abstract

**Background:**

Tacrolimus is widely used as an immunosuppressant in the management of refractory nephrotic syndrome. Although effective, it may occasionally lead to rare but serious adverse effects such as posterior reversible leukoencephalopathy syndrome (PRES). PRES has traditionally been associated with hypertension and elevated drug concentrations.

**Case presentation:**

In the present study, we describe the case of a 10-year-old Chinese girl who was diagnosed with steroid-resistant nephrotic syndrome (SRNS) and pathologically confirmed to have minimal change nephropathy. Following 40 days of full-dose glucocorticoid therapy with inadequate improvement in proteinuria, tacrolimus was initiated at 1.5 mg twice daily (0.09 mg/kg/day). Neurological symptoms, including headache and nausea, developed 18 h after the first dose—before steady-state drug levels were reached. Within 24 h, hypertension emerged, and magnetic resonance imaging (MRI) revealed abnormal signals in the bilateral parietal cortical and subcortical regions, consistent with PRES. Tacrolimus was immediately discontinued, and the patient was treated with nifedipine, low-dose furosemide, and vitamin B6. Symptoms resolved within 48 h, and blood pressure normalized. Immunosuppressive therapy was subsequently switched to mycophenolate mofetil (MMF). Follow-up brain MRI at three months demonstrated complete resolution of the detected abnormalities.

**Conclusion:**

Tacrolimus-associated PRES may occur very early in treatment, even before stable drug concentrations are achieved. Vigilant clinical monitoring, prompt recognition of neurological symptoms, and timely intervention are critical to avoid long-term sequelae.

## Introduction

Nephrotic syndrome (NS) is a clinical condition characterized by massive proteinuria, hypoalbuminemia, hyperlipidemia, and edema. Its pathogenesis is multifactorial, involving immunologic dysregulation, genetic predisposition, and environmental influences. Minimal change nephropathy is the most common pathological subtype in children. Glucocorticoids remain the cornerstone of therapy, and the majority of patients respond favorably. However, a subset of children develop steroid resistance, leading to persistent proteinuria, impaired quality of life, and adverse prognosis (1). Managing steroid-resistant nephrotic syndrome (SRNS) is challenging. While immunosuppressive agents and biologics have improved outcomes for some patients, their use is limited by variable efficacy and significant adverse effects (2, 3).

Based on evidence-based guidelines, tacrolimus, a calcineurin inhibitor, is recommended as a first-line immunosuppressant for SRNS (1). Its antiproteinuric activity is believed to stem from hemodynamic changes that reduce renal blood flow, inhibition of calcineurin-mediated podocyte protein degradation, and stabilization of the actin cytoskeleton (1). Despite its effectiveness, tacrolimus is associated with adverse effects, among which reversible posterior leukoencephalopathy syndrome (PRES) represents a rare but serious neurotoxic event (4).

Here, we report the case of a 10-year-old girl who presented with steroid-resistant minimal change nephropathy and subsequently developed PRES soon after starting tacrolimus. Notably, the neurological complications occurred rapidly, within hours of treatment initiation, and were followed by the onset of hypertension. The child responded well to discontinuation of tacrolimus and supportive therapy, with full recovery documented on follow-up imaging. This case underscores the need for clinicians to maintain a high index of suspicion for PRES in pediatric NS patients receiving tacrolimus, especially in the presence of multiple predisposing factors.

## Case presentation

A 10-year-old girl was admitted with a 40-day history of foamy urine. Initially, she had developed unexplained periorbital and lower limb edema and was evaluated at a local hospital. Laboratory investigations demonstrated marked proteinuria, hypoalbuminemia, and hyperlipidemia. Autoimmune serological tests and hepatitis B surface antigen were negative. The patient had no rash or arthralgia, no history of hypertension, and no relevant family history of kidney disease, neurological disorders, or hypertension, effectively excluding secondary causes of nephrotic syndrome. On this basis, she was diagnosed with primary nephrotic syndrome and initiated on full-dose oral prednisone (60 mg/day) in combination with captopril. She had not received any recent intravenous methylprednisolone pulse therapy. The edema subsided, but after 40 days, proteinuria persisted, prompting referral to our center on August 30, 2023. On admission, she continued to exhibit nephrotic-range proteinuria (urine protein 3+, red blood cells 0/µL, urine albumin/creatinine ratio 318.8 mg/mmol, 24-hour urine protein 1.7 g, urine β2-microglobulin/creatinine 26.3 µg/mmol, urinary N-acetyl-β-D-glucosaminidase 4.7 U/L). Lipid profile showed total cholesterol 9.88 mmol/L (normal: 2.30–5.20), triglycerides 2.69 mmol/L (0.56–1.70), and LDL 5.06 mmol/L (1.20–3.30). Serum albumin was 35.8 g/L, with normal liver function and creatinine (39.6 µmol/L).

On admission, the patient's blood pressure was 115/70 mmHg. Physical examination demonstrated a Cushingoid appearance, normal growth parameters (height 140 cm, weight 33 kg), and no evidence of edema. A subsequent renal biopsy confirmed minimal change nephropathy. In the setting of persistent proteinuria, she was diagnosed with steroid-resistant nephrotic syndrome. After excluding infection, treatment with immunosuppressive agents or biologic therapy, such as rituximab, was recommended. However, the family declined biologic therapy because of concerns regarding potential adverse effects and consented to tacrolimus treatment.

**Figure 1:**
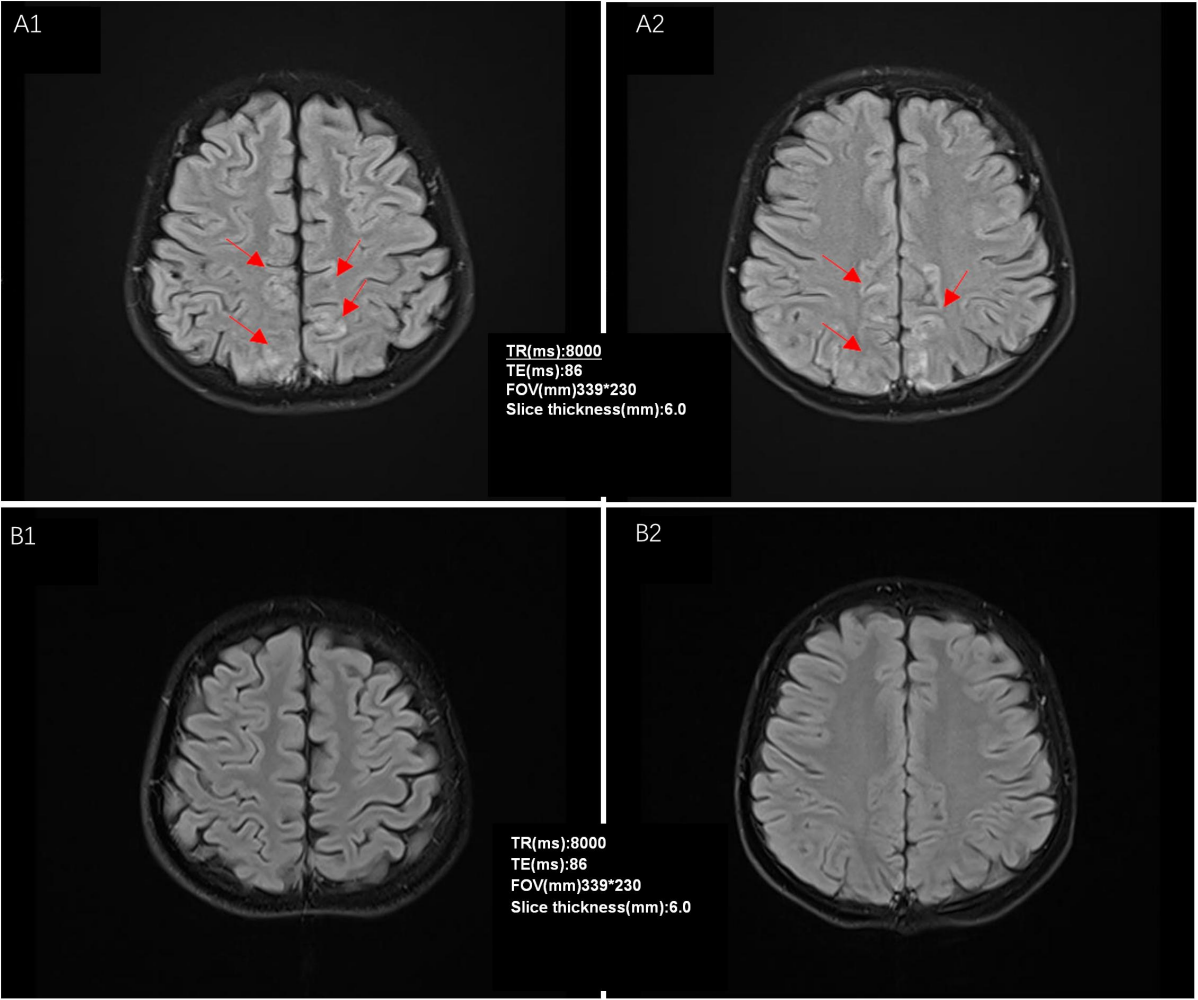
Patient's head imaging assessment. A1 and A2 are bilateral parietal lobe cortical and subcortical patchy slightly high signal shadows on T2-FLAIR images at the onset of the disease. B1 and B2 in the figure show that the abnormal signal shadows in the bilateral parietal lobe cortex and subcortex on T2-FLAIR images have completely disappeared when reexamined 3 months after the onset of the disease.

**Figure 2:**
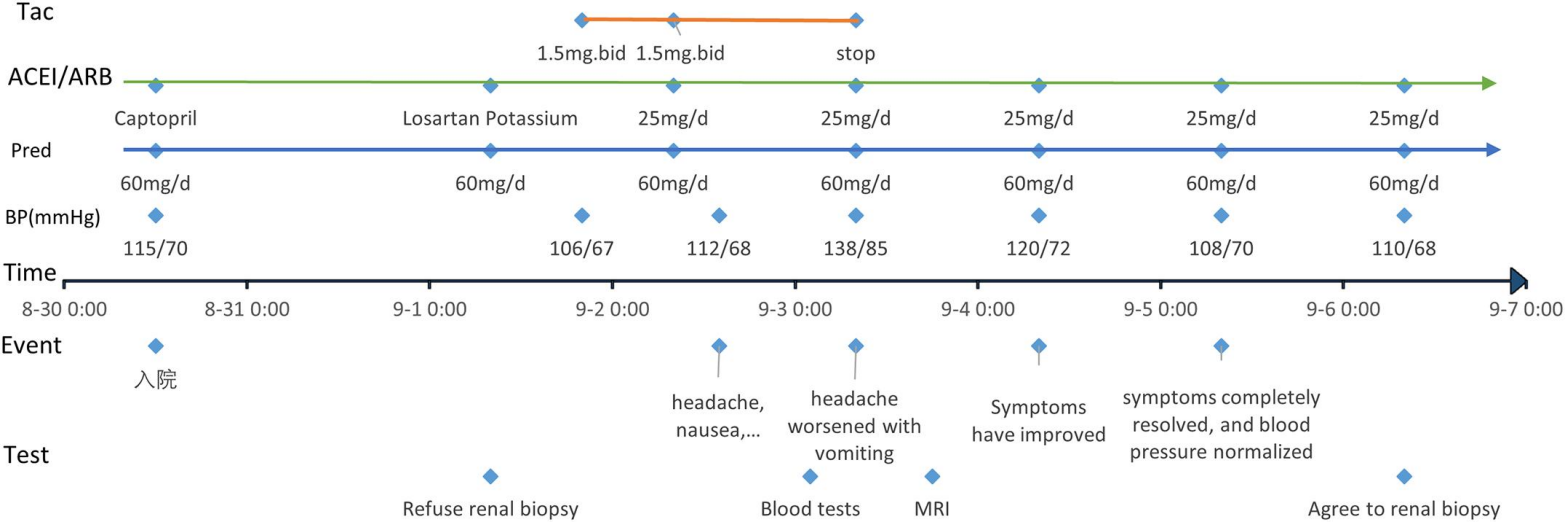
Clinical interventions vs. adverse reaction onset time chart.

At 8:00 pm on September 1, 2023, she received her first dose of tacrolimus (1.5 mg orally twice daily; 0.09 mg/kg/day), at which time her blood pressure was 106/67 mmHg. Eighteen hours later, she developed a headache, nausea, and malaise, with a blood pressure of 112/68 mmHg. By 8:00 am on September 3, her headache had worsened and was accompanied by vomiting, and her blood pressure had increased to 138/95 mmHg. Laboratory evaluation showed a serum albumin level of 31.5 g/L, normal procalcitonin and complete blood count results, normal liver function tests, normal serum sodium, potassium, and calcium levels, and normal disseminated intravascular coagulation parameters. All medications were administered orally, and there was no clinical evidence of fluid overload. Ophthalmological evaluation showed normal intraocular pressure. Emergent MRI revealed abnormal signals in the bilateral parietal cortical and subcortical regions (Figure 1), consistent with PRES. Tacrolimus was discontinued immediately. Supportive treatment included nifedipine and low-dose furosemide to control blood pressure and cerebral edema, along with vitamin B6 for neuroprotection. Within 48 h, symptoms completely resolved, and blood pressure normalized. Figure 2 depicts the chronological sequence of events throughout the entire course. She was transitioned to mycophenolate mofetil for ongoing immunosuppression. At the three-month follow-up, repeat MRI demonstrated full resolution of these abnormalities (Figure 1).

## Discussion

In this case, the child's clinical presentation and laboratory findings were consistent with nephrotic syndrome. Despite receiving standard glucocorticoid therapy, heavy proteinuria persisted, fulfilling the criteria for SRNS. Following the initiation of tacrolimus, the patient developed acute neurological symptoms and was subsequently diagnosed with reversible PRES Prompt recognition of the condition, discontinuation of the offending drug, and rapid initiation of supportive measures, including antihypertensive and neuroprotective therapy, prevented further complications and resulted in complete neurological recovery. This case underscores the importance of vigilance for adverse drug reactions during immunosuppressive therapy in pediatric patients and highlights the necessity of immediate intervention when neurological symptoms appear.

Five established high-risk factors for the development of PRES were present in this patient (5–8): (1) tacrolimus exposure; (2) steroid-induced hypertension; (3) an active nephrotic state complicated by hypoproteinemia; (4) concomitant use of ACE inhibitors and ARB agents; and (5) female sex. After withdrawal of tacrolimus, the key variable under consideration, the patient's neurological symptoms resolved, and blood pressure normalized. Accordingly, neurological manifestations attributable to steroid-related hypertension, the active nephrotic state with hypoalbuminemia, or ACEI/ARB-related adverse effects were excluded, supporting the conclusion that PRES in this case was mediated by tacrolimus. Tacrolimus, a calcineurin inhibitor, is increasingly used in children with nephrotic syndrome, particularly in steroid-resistant or steroid-dependent cases (1). While it is effective in achieving disease control, tacrolimus is also associated with uncommon but potentially serious adverse reactions, one of which is PRES, a neurotoxic complication linked to calcineurin inhibitors. PRES is characterized by vasogenic cerebral edema and is frequently reported in the context of hypertension, preeclampsia, renal dysfunction, or immunosuppressive therapy, especially among solid organ transplant recipients (4, 9). Clinically, affected individuals may present with headache, nausea, vomiting, seizures, disturbances in vision, altered levels of consciousness, and, in some instances, focal neurological deficits (3, 10). With respect to imaging studies PRES typically manifests as hyperintense lesions on T2-weighted and FLAIR sequences, most often involving the parietal and occipital lobes, although other brain regions can also be affected (5). Because the condition is often reversible with prompt intervention, such as withdrawal of the offending agent and control of blood pressure, early recognition is essential. Conversely, delayed diagnosis and treatment may lead to irreversible cerebral injury (5).

Previous reports have shown that tacrolimus-associated neurotoxicity can develop from several days to several years after treatment initiation, with the majority of cases reported in transplant populations. In a systematic review, Paige Verona compared the timing of PRES onset across reported cases (4). The reported time to onset may be underestimated, however, since post-transplant confounding factors complicate interpretation (4). Pharmacokinetic studies show that tacrolimus has a half-life of approximately 10–12 h (4), with steady-state plasma levels reached after 5–7 days of continuous administration. In our patient, who was not a transplant recipient, neurological symptoms developed strikingly early—arising within 18 h of tacrolimus exposure, corresponding to approximately one half-life, before steady-state concentrations were achieved. We propose that this unusually rapid onset may have been related to fluctuations in drug levels and individual differences in metabolism that provoked a neurovascular response. Several mechanisms have been proposed to explain the pathophysiology of tacrolimus-induced PRES. First, polymorphisms in the *CYP3A5* and *ABCB1* genes may impair the function of the ATP-dependent efflux transporter P-glycoprotein (P-gp) at the blood–brain barrier (BBB), thereby reducing the clearance of tacrolimus from neural tissue and permitting its accumulation in the central nervous system (4, 11, 12). Second, tacrolimus itself may downregulate P-gp expression, increase vascular permeability, and induce apoptosis in cerebral microvascular endothelial cells (MBEC4), leading to disruption of barrier integrity and vasogenic edema (4). Third, elevated tacrolimus concentrations in neural tissue may contribute to mitochondrial dysfunction, promote the generation of reactive oxygen species, and directly damage neuronal structures (4, 13, 14).

Hypertension is commonly observed in PRES, and many studies consider hypertensive crisis with cerebral hyperperfusion to be a key pathogenic driver (3, 15, 16). However, the association is not universal, as approximately 30% of patients present with normal or only mildly elevated blood pressure (15). In our case, the absence of post-transplant confounding factors and the fact that neurological symptoms preceded the onset of hypertension by nearly 24 h suggest a blood pressure–independent mechanism. This observation aligns with an alternative hypothesis, proposed in a minority of studies, which attributes the initiation of PRES to direct endothelial injury from circulating endogenous or exogenous toxins. According to this “toxin-endothelial injury” model, disruption of the vascular barrier leads to leakage, edema, endothelial activation, release of vasoactive mediators, and increased vascular permeability. Within this framework, hypertension is interpreted as a secondary event arising from primary endothelial dysfunction rather than the initiating cause of PRES (17).

Interestingly, most reported cases of tacrolimus-associated neurotoxicity have occurred within the therapeutic drug concentration range (4). This seems inconsistent with the traditional intuitive understanding of “drug intoxication” or “excessively high concentration”. Currently, several potential mechanisms are as follows: First, individual differences in the blood-brain barrier (BBB) and altered permeability are the core factors (4, 18, 19). When BBB function is impaired or P-glycoprotein (P-gp) activity is reduced, the drug concentration in cerebrospinal fluid (CSF) or brain parenchyma may increase abnormally even if the blood concentration is within the therapeutic window. Second, therapeutic drug monitoring (TDM) of blood concentration has inherent limitations. The measured blood concentration reflects the total drug concentration in peripheral blood (including the protein-bound fraction), whereas it is the unbound (free) drug that crosses the BBB and exerts neurotoxic effects (20). Individual variations in protein binding rate and metabolic enzyme activity (e.g., CYP3A4/5) can lead to differences in free drug concentration and subsequent brain tissue exposure. Blood drug concentration cannot directly reflect the penetration and accumulation of the drug in the central nervous system (CNS) (20). Its established safe range is not equivalent to the safety threshold for CNS toxicity (20). Therefore, for certain susceptible individuals, concentrations deemed “within the therapeutic range” may still be excessively high. Third, individual vascular endothelial cells may exhibit abnormal sensitivity to tacrolimus-induced toxicity (4, 19), which can result in functional impairment even at conventional concentrations. In light of this, we recommend that for patients receiving tacrolimus therapy, the possibility of PRES should be immediately considered upon the onset of any neurological symptoms, regardless of whether their blood drug concentration falls within the target range.

In the majority of cases, management primarily involved adjusting the immunosuppressive regimen, most commonly switching from tacrolimus to cyclosporine or to alternative agents, together with supportive treatment for neurological manifestations. Following these interventions, most patients experienced complete symptom resolution (4). The role of B-vitamin supplementation in posterior reversible encephalopathy syndrome (PRES) remains controversial. However, several studies have suggested that vitamin B6 may exert neuroprotective effects against central nervous system injury through multiple mechanisms (21–23): including: (1) attenuation of endothelial oxidative stress; (2) modulation of glutamatergic neurotransmission; and (3) potential stabilization of the blood–brain barrier. Although the supporting evidence is still evolving, these mechanisms are highly relevant to the underlying pathophysiology of PRES.

## Conclusion

This case represents, to our knowledge, the earliest reported onset of tacrolimus-induced PRES, with neurological symptoms occurring only 18 h after the first dose and before attainment of steady-state drug levels. Prompt recognition, early neuroimaging, and timely discontinuation of tacrolimus, together with targeted therapeutic interventions, led to complete clinical resolution and a favorable prognosis.

Importantly, this case illustrates that PRES may occur independently of significant hypertension, lending support to the “toxin–endothelial dysfunction–PRES–secondary hypertension” model rather than the traditional “hypertension–PRES” paradigm. These findings emphasize the need for heightened clinical awareness when initiating tacrolimus in pediatric nephrotic syndrome. Continuous monitoring of both neurological status and blood pressure is essential, and we recommend implementing a “neurological symptoms plus blood pressure surveillance” protocol to facilitate early detection of tacrolimus-related PRES.

## Data Availability

The original contributions presented in the study are included in the article/Supplementary Material, further inquiries can be directed to the corresponding author.
